# Tonic activin signaling shapes cellular and synaptic properties of CA1 neurons mainly in dorsal hippocampus

**DOI:** 10.1016/j.isci.2023.108001

**Published:** 2023-09-21

**Authors:** Marc Dahlmanns, Maria Jesus Valero-Aracama, Jana Katharina Dahlmanns, Fang Zheng, Christian Alzheimer

**Affiliations:** 1 Institute of Physiology and Pathophysiology, Friedrich-Alexander-Universität Erlangen-Nürnberg, 91054 Erlangen, Germany

**Keywords:** Neuroscience, Cellular neuroscience

## Abstract

Dorsal and ventral hippocampus serve different functions in cognition and affective behavior, but the underpinnings of this diversity at the cellular and synaptic level are not well understood. We found that the basal level of activin A, a member of the TGF-β family, which regulates hippocampal circuits in a behaviorally relevant fashion, is much higher in dorsal than in ventral hippocampus. Using transgenic mice with a forebrain-specific disruption of activin receptor signaling, we identified the pronounced dorsal-ventral gradient of activin A as a major factor determining the distinct neurophysiologic signatures of dorsal and ventral hippocampus, ranging from pyramidal cell firing, tuning of frequency-dependent synaptic facilitation, to long-term potentiation (LTP), long-term depression (LTD), and de-potentiation. Thus, the strong activin A tone in dorsal hippocampus appears crucial to establish cellular and synaptic phenotypes that are tailored specifically to the respective network operations in dorsal and ventral hippocampus.

## Highlights

• Basal activin A level is high in dorsal, and low in ventral hippocampus
• Activin gradient shapes cell firing and synapse function along hippocampal axis
• Activin A specifies range of frequency facilitation in dorsal hippocampus
• Tonic activin signaling regulates LTP and LTD solely in dorsal hippocampus

## Graphical abstract

**Figure:**
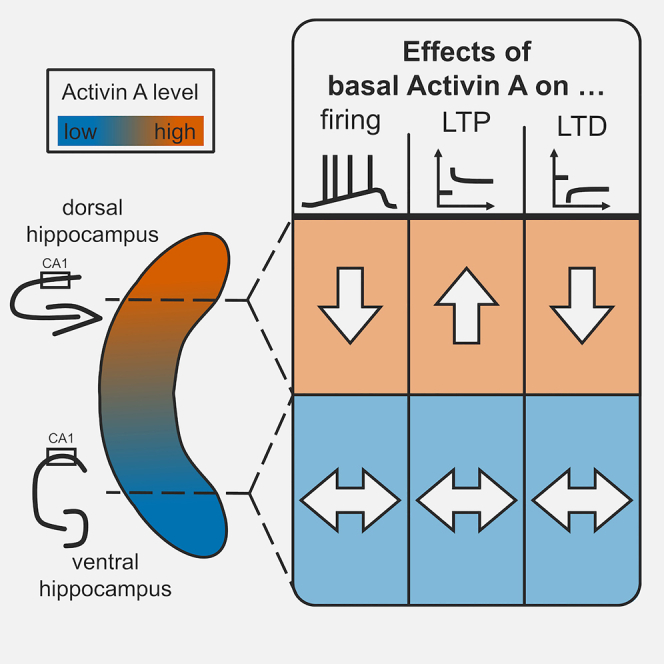

## Introduction

It is increasingly recognized that the hippocampus is not a uniform structure, but rather serves distinct functions along its longitudinal (dorsal-ventral) axis. Whereas the dorsal part of hippocampus is involved mainly in cognitive and memory functions, with a particular emphasis on spatial orientation in rodents, the ventral part is embedded in a network that processes emotions and regulates affective behavior.^1^^,^^2^^,^^3^ To enable their functional diversity, dorsal (DH) and ventral hippocampus (VH) are endowed with a different mix of network, synaptic, cellular, and molecular properties. For example, at the single-cell level, ventral CA1 pyramidal cells are more excitable than their dorsal counterparts.^4^^,^^5^^,^^6^^,^^7^^,^^8^^,^^9^ Regarding basal synaptic transmission along the longitudinal axis, inconsistent findings have been reported,^10^^,^^11^^,^^12^^,^^13^ whereas it was unanimously shown that short-term plasticity (STP) is more pronounced in dorsal CA1 compared to ventral CA1.^11^^,^^12^^,^^14^^,^^15^^,^^16^^,^^17^ Long-term potentiation (LTP) was also more prominent in dorsal CA1.^7^^,^^9^^,^^18^^,^^19^^,^^20^ It remains to be determined, though, whether a similar regional segregation holds for long-term depression (LTD), given that this phenomenon has been largely studied in dorsal hippocampus.

Whereas studies at various levels of complexity have identified a number of marked differences between DH and VH, the mechanisms underlying their distinct behavioral, neurophysiological, and molecular profiles are far less clear. Here, we examined cellular excitability and synaptic plasticity in dorsal and ventral CA1 pyramidal cells to examine the hypothesis that activin A, a member of the transforming growth factor (TGF)-β family, is involved in specifying their distinct neurophysiological phenotypes.

Activin A is a homodimeric protein formed by two disulfide-linked βA subunits, which are encoded by the gene *Inhba*. In addition to its well-established role as neurotrophic and neuroprotective factor in brain development and acute brain damage, respectively,^21^^,^^22^^,^^23^^,^^24^ activin A emerged as a “master molecule” that tunes neuronal properties in a behaviorally relevant fashion.^25^ This concept also offers new insights into the pathogenesis and therapy of neuropsychiatric diseases.^26^ For example, activin signaling has been implicated in anxiety disorders, major depression, alcohol consumption, and drug addiction.^26^^,^^27^ On the physiological side, activin A has been shown to regulate GABA_A_ receptor- and GABA_B_ receptor-mediated inhibition, to modulate the response of GABA_A_ receptors to benzodiazepines and alcohol, to control cellular excitability through modulation of G protein-activated inwardly rectifying K^+^ (GIRK) currents, and to optimize the performance of glutamatergic synapses as a means to promote learning and memory.^25^^,^^28^^,^^29^^,^^30^^,^^31^^,^^32^^,^^33^

Activin A acts through a tetrameric receptor complex of type I and II receptors, in which activin receptor IB (ActRIB) triggers downstream signaling pathways. To decipher the relative contribution of activin A to dorsal-ventral disparities in the respective cellular and synaptic properties, we made use of an established mouse line that expresses a dominant-negative mutant of ActRIB under the control of the CaMKII-α promoter (dnActRIB), thus achieving a forebrain-specific disruption of activin signaling without interfering with the most critical period of postnatal development.^28^ Based on our findings with hippocampi from wild type (wt) and dnActRIB mice, we introduce activin A as an essential factor to regulate neural properties of the hippocampus in a region-specific fashion along its longitudinal axis.

## Results

### Excitability of CA1 pyramidal cells increases from dorsal to ventral pole of hippocampus

**Figure 1:**
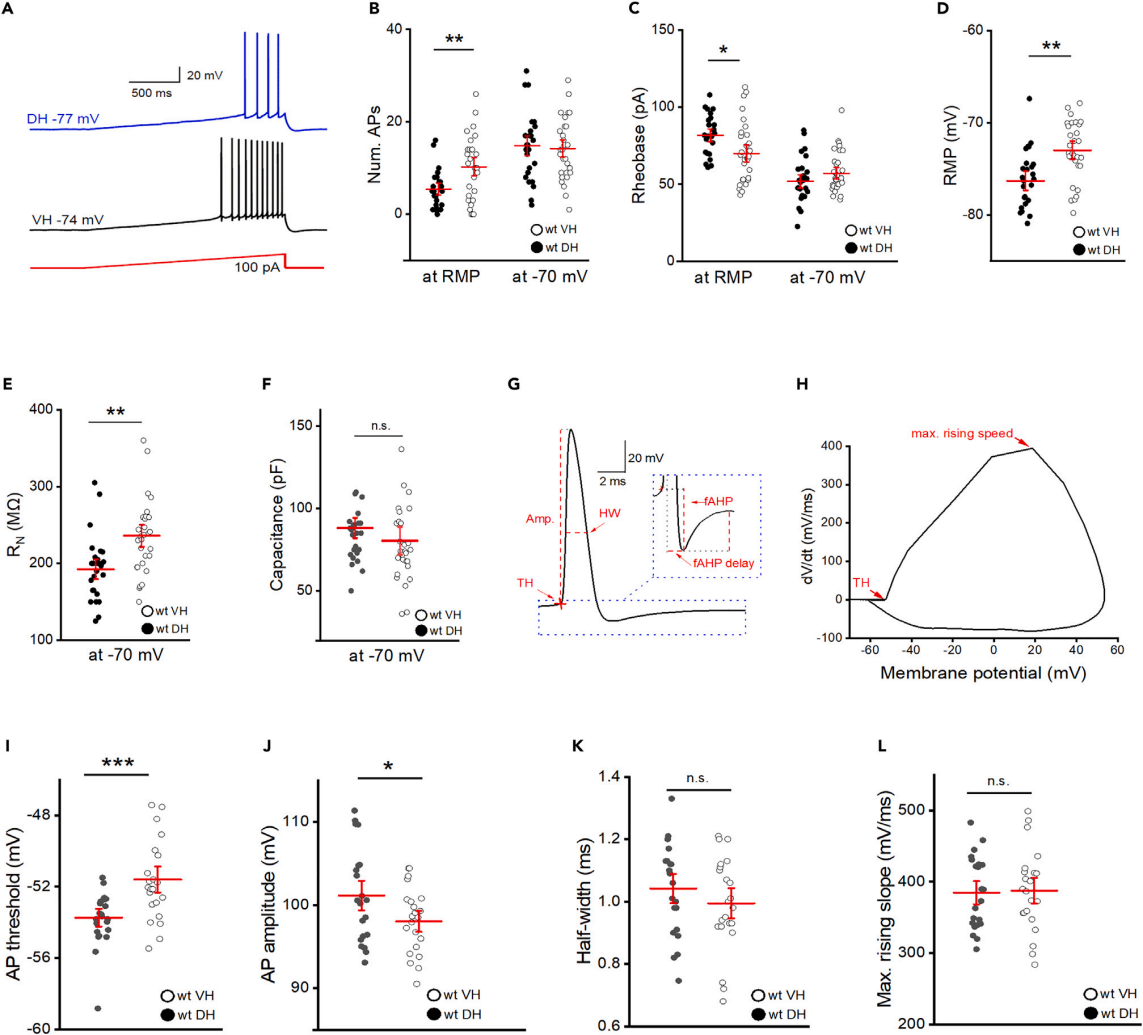
Excitability is higher in ventral than in dorsal CA1 pyramidal cells that display differences in passive membrane properties (A) Representative firing responses of dorsal (blue) and ventral (black) CA1 pyramidal cell to ramp-shaped current injection (0–100 pA) from resting membrane potential (RMP). (B and C) Summary of ramp-evoked action potential (AP) firing and rheobase current for dorsal (filled circles) and ventral cells (open circles), from RMP (left) and from −70 mV (right), shown as individual data points with mean ± SEM (red). (D–F) RMP (D), input resistance (R_N_, E), and capacitance (F) of dorsal and ventral cells. (G–L) AP parameters were analyzed based on the first action potential elicited by current injection from RMP. Trace example of a single AP in G indicates how spikes were gauged. Inset depicts spike after-potential at higher magnification. H shows the first derivative of AP voltage trajectory over time, plotted as function of membrane potential. Sites of AP threshold and maximum rising speed are indicated over the trace. AP threshold (I), amplitude (J), half-width (K), and maximum rising slope (L) are depicted as individual data points from dorsal cells (filled circles) and ventral cells (open circles), and as mean ± SEM (red). Statistical comparisons were performed using an unpaired, two-tailed Student’s *t* test at α = 0.05. ∗p < 0.05, ∗∗∗p < 0.001.

To examine whether the firing behavior of CA1 neurons changes along the longitudinal axis of the hippocampus, we performed whole-cell current-clamp recordings from CA1 pyramidal cells in slices containing either the dorsal or the ventral portion of the hippocampus. As illustrated in Figure 1A, depolarizing current ramps from 0 to 100 pA were used to elicit action potential (AP) firing, starting from resting membrane potential (RMP). The firing propensity was higher in ventral CA1 pyramidal cells (wt VH, 10.14 ± 1.29 APs per ramp, n = 29) than in dorsal CA1 cells (wt DH, 5.29 ± 0.87 APs per ramp, n = 24; p = 0.004; Figure 1B). Rheobase measurements revealed that the current required to elicit the first AP during gradual depolarization was lower in ventral (70.89 ± 3.68 pA) than in dorsal pyramidal cells (82.32 ± 2.69 pA; p = 0.019, Figure 1C). However, the difference in firing vanished when depolarizing ramps started from membrane potential preset to −70 mV by current injection (Figures 1B and 1C).

Consistent with previous reports,^4^^,^^5^^,^^6^^,^^7^^,^^8^ passive electrical properties exhibited significant asymmetries between ventral and dorsal neurons. Specifically, RMP was more negative in dorsal CA1 (−76.08 ± 0.62 mV) than in ventral CA1 (−73.05 ± 0.60 mV; p = 0.001, Figure 1D), input resistance (R_N_) was higher in ventral than in dorsal cells (measured at −70 mV, ventral CA1: 234.21 ± 8.99 MΩ; dorsal CA1: 185.69 ± 7.96 MΩ; p = 0.001, Figure 1E), while capacitance was comparable in both groups (ventral CA1: 77.84 ± 4.07 pF; dorsal CA1: 83.81 ± 2.86 pF; p = 0.320, Figure 1F) (Table 1).

**Table 1:** Passive membrane properties of dorsal and ventral CA1 pyramidal cells from wt and dnActRIB mice

	wt DH (n = 24)	wt VH (n = 29)	dnActRIB DH (n = 17)	dnActRIB VH (n = 21)
RMP (mV)	−76.08 ± 0.62	−73.05 ± 0.60 ∗∗	−74.42 ± 0.77	−73.50 ± 0.54
Input resistance (MΩ)	185.69 ± 7.96	234.21 ± 8.99 ∗∗	235.44 ± 9.94	265.57 ± 13.16
Capacitance (pF)	83.81 ± 2.86	77.84 ± 4.07	64.30 ± 2.95	71.86 ± 3.92

Asterisks (∗) indicate significant dorsal-ventral differences. Statistical analysis was performed using unpaired, two-tailed student’s t-test. ∗∗ p < 0.01.

After having established distinct passive properties of dorsal and ventral CA1 neurons as a sizable factor for the dorsal-ventral gradient in cellular firing, we took a closer look at AP waveforms. A detailed analysis of the first AP in a train of discharges elicited by ramp depolarization from rest (Figures 1G–1L; Table 2) showed that, first, AP threshold was more depolarized in dorsal CA1 than in ventral CA1 (Figure 1I), in line with previous reports,^4^^,^^5^^,^^8^ and, second, that AP amplitude was significantly higher in dorsal CA1 than in ventral CA1 (Figure 1J). By contrast, the following parameters did not differ between groups: spike half-width (Figure 1K), maximum speed during AP upstroke (Figure 1L), amplitude and delay of fast afterhyperpolarization (fAHP) (Table 2).

**Table 2:** Action potential parameters of dorsal and ventral CA1 pyramidal cells from wt and dnActRIB mice

	wt DH (n = 23)	wt VH (n = 22)	dnActRIB DH (n = 12)	dnActRIB (n = 16)
Amplitude (mV)	101.16 ± 1.15	98.14 ± 0.84 ∗	100.57 ± 1.04	93.62 ± 1.56 ∗∗, #
Threshold (mV)	−53.89 ± 0.31	−51.73 ± 0.48 ∗∗∗	−54.21 ± 0.59	−52.05 ± 0.96 ∗
Half-width (ms)	1.03 ± 0.03	0.99 ± 0.03	0.91 ± 0.05 #	1.09 ± 0.04 ∗
Max. rise slope (mV/ms)	384.86 ± 10.40	387.48 ± 12.16	457.47 ± 22.71 ##	321.35 ± 17.35 ∗∗∗, ##
fAHP (mV)	7.47 ± 0.52	7.69 ± 0.67	7.89 ± 0.77	7.28 ± 0.71
fAHP delay (ms)	2.46 ± 0.07	2.42 ± 0.07	2.14 ± 0.07 ##	2.69 ± 0.10 ∗∗, #

Asterisks (∗) indicate significant dorsal-ventral differences. Hashes (#) indicate differences between wt and dnActRIB genotypes. Statistical analysis was performed using unpaired, two-tailed student’s t-test. ∗, # p < 0.05; ∗∗, ## p < 0.01; ∗∗∗ p < 0.001.

### Dorsal-ventral difference of activin A tunes cellular excitability in a region-specific fashion

**Figure 2:**
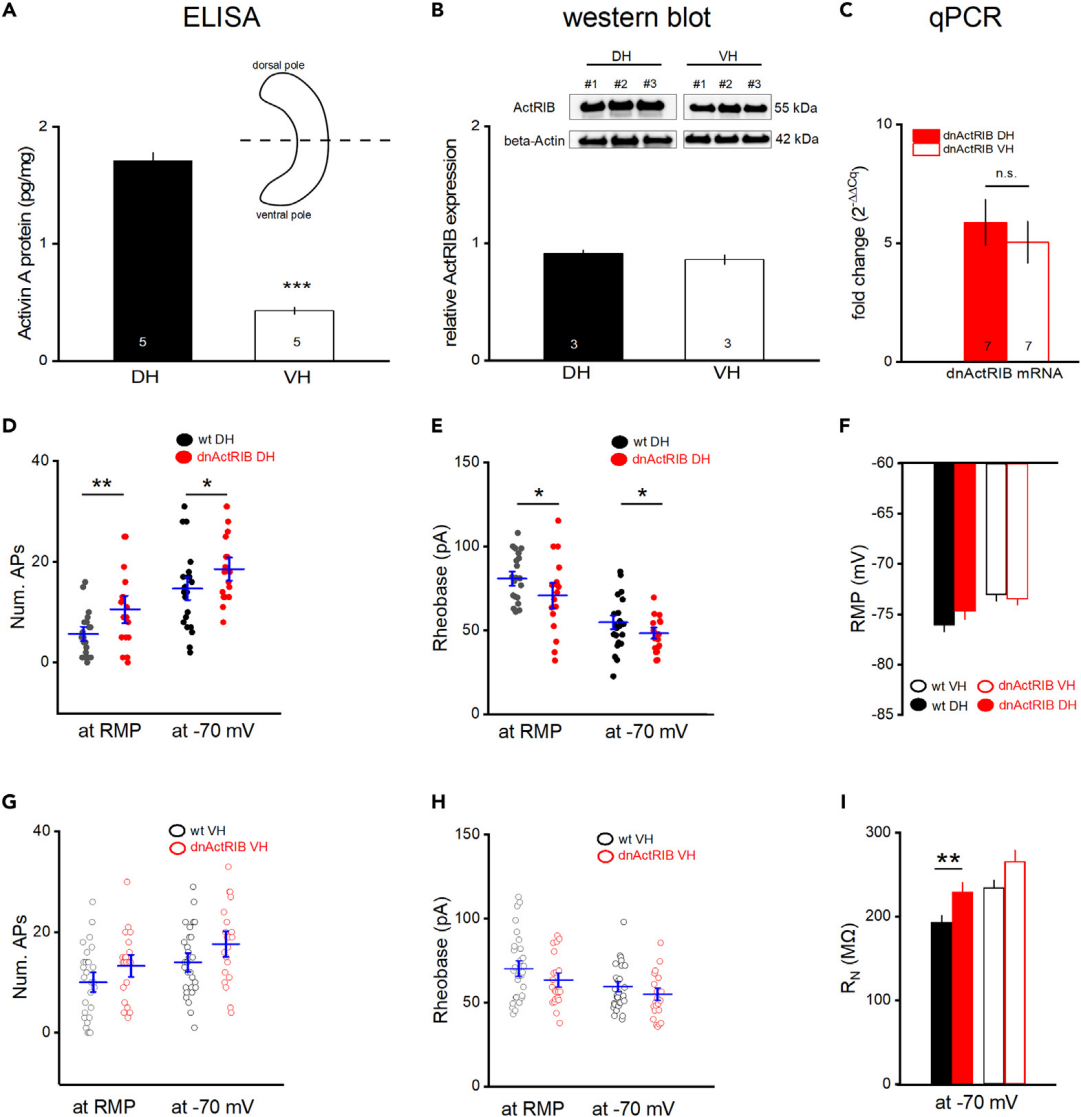
Elimination of activin receptor signaling abrogates dorsal-ventral difference in ramp-evoked AP firing of CA1 neurons (A) Hippocampus was halved (inset) and activin A protein levels were determined with use of ELISA for dorsal and ventral hippocampus (DH and VH). (B) Activin receptor IB is equally expressed in DH and VH. Western Blots were performed using antibodies against activin receptor IB (ActRIB, 55 kDa) and β-actin (42 kDa). Representative ActRIB- and β-actin-stained blots are shown above. ActRIB-bands were stained and imaged first, followed by β-actin staining and imaging. Relative optical density was calculated based on β-actin-normalized gray scale values using ImageJ. (C) RT-qPCR shows equal levels of dnActRIB mRNA in DH and VH of mutant mice. (D, E, G, and H) Firing responses to depolarizing ramps were compared between wt (black) and dnActRIB (red) dorsal (D and E) and ventral (G and H) CA1 pyramidal cells from RMP (left) and from −70 mV (right). (F and I) Histograms compare RMP (F) and membrane input resistance (at −70 mV; I) of dorsal and ventral CA1 neurons from both genotypes. Values are given as mean ± SEM. Statistical comparisons were performed using an unpaired, two-tailed Student’s *t* test at α = 0.05. ∗p < 0.05, ∗∗p < 0.01. ∗∗∗p < 0.001.

In previous work, we found that activin receptor signaling modulates firing of CA1 pyramidal cells, as well as firing of granule cells in the dentate gyrus.^32^^,^^33^ Since all those studies were performed on neurons from DH, we wondered how activin signaling would affect excitability in ventrally located neurons. In a first step, we quantified the expression of activin A, the best characterized and most abundant isoform in the brain, together with ActRIB, the main signal-transducing receptor, separately for DH and VH. Importantly, we found a much higher level of activin A in DH (1.71 ± 0.07 pg/mg, n = 5) than in VH (0.43 ± 0.03 pg/mg, n = 5; p = 1.94e^−7^, Figure 2A). By contrast, ActRIB was not differentially expressed (ActRIB/β-Actin; wt DH: 0.91 ± 0.02, n = 3; wt VH: 0.86 ± 0.04, n = 3, p = 0.30; Figure 2B). This uniform expression pattern holds also for the dominant-negative mutant of activin receptor IB (dnActRIB) that, by disrupting activin receptor signaling in a forebrain-specific fashion, serves as a valuable tool to elucidate the function of endogenous activin in normal and diseased brain.^25^^,^^28^^,^^30^ As shown in Figure 2C, mRNA abundance of the dnActRIB transgene does not display any dorsal-ventral gradient (2^−ΔΔCt^ for dnActRIB; dnActRIB DH: 5.88 ± 0.97, n = 7; dnActRIB VH: 5.04 ± 0.89, n = 7, p = 0.56). Also, transgene expression does not alter mRNA abundance of the remaining wt-ActRIB allele in a DH- or VH-specific fashion (2^−ΔΔCt^ for wt ActRIB in dnActRIB slices; dnActRIB DH: 1.07 ± 0.17, n = 7; dnActRIB VH: 1.10 ± 0.07, n = 7; p = 0.87).

Using slices from dnActRIB mice, we asked next whether the strikingly asymmetric distribution of activin A along the longitudinal axis is instrumental for maintaining the difference in firing behavior between CA1 neurons from dorsal and VH. As can be seen on Figures 2D, 2E, 2G, and 2H, the mutation increased excitability selectively in dorsal CA1 pyramidal cells (dnActRIB dorsal CA1, n = 17: number of APs, 10.41 ± 1.85, p = 0.009 vs. wt dorsal CA1; rheobase 70.86 ± 5.46 pA; p = 0.047 vs. wt dorsal CA1), which exhibited now a discharge pattern indistinguishable from that of CA1 pyramidal cells in VH, both in terms of rheobase and AP number per ramp from rest (dnActRIB ventral CA1, n = 21: number of APs 13.38 ± 1.46 APs, p = 0.104 vs. wt ventral CA1, p = 0.210 vs. dnActRIB dorsal CA1; rheobase 62.93 ± 3.33 pA, p = 0.131 vs. wt ventral CA1, p = 0.205 vs. dnActRIB dorsal CA1). The same dichotomy was observed, when depolarizing ramps were delivered from −70 mV (Figures 2D, 2E, 2G, and 2H). Notably, although no significant difference with respect to wt cells was observed in RMP of mutant dorsal CA1 (−74.42 ± 0.77 mV; p = 0.100 vs. wt dorsal CA1) and ventral CA1 (−73.50 ± 0.54 mV; p = 0.603 vs. wt ventral CA1; Figure 2F), the dorsal-ventral difference in RMP in wt hippocampi (Figure 1D) was leveled out in the mutant preparation (p = 0.147). By contrast, the transgene increased R_N_ dorsally (at −70 mV, dnActRIB dorsal CA1: 235.44 ± 9.94 MΩ; p = 0.002 vs. wt dorsal CA1) and tended to modulate it ventrally (dnActRIB ventral CA1: 265.57 ± 13.16 MΩ, p = 0.060 vs. wt dorsal CA1; Figure 2I). These findings strongly suggest that the much higher basal level of activin A in dorsal than VH is essential to regulate cellular excitability in a region-specific fashion.

With respect to the characteristic features of AP waveforms in dorsal and ventral CA1 neurons, we found that the disruption of activin receptor signaling was associated with a reduction in peak amplitude in cells from ventral regions. Interestingly, the kinetics of upstroke were slowed dorsally and speeded ventrally, and the repolarization were speeded dorsally and slowed in ventral neurons (Table 2). It remains to be determined whether the here observed changes in ventral APs is a direct consequence of the elimination of activin signaling or rather represents an adaptive process.

### Effects of activin signaling on Schaffer collateral-CA1 synapses at low and high stimulation frequencies

**Figure 3:**
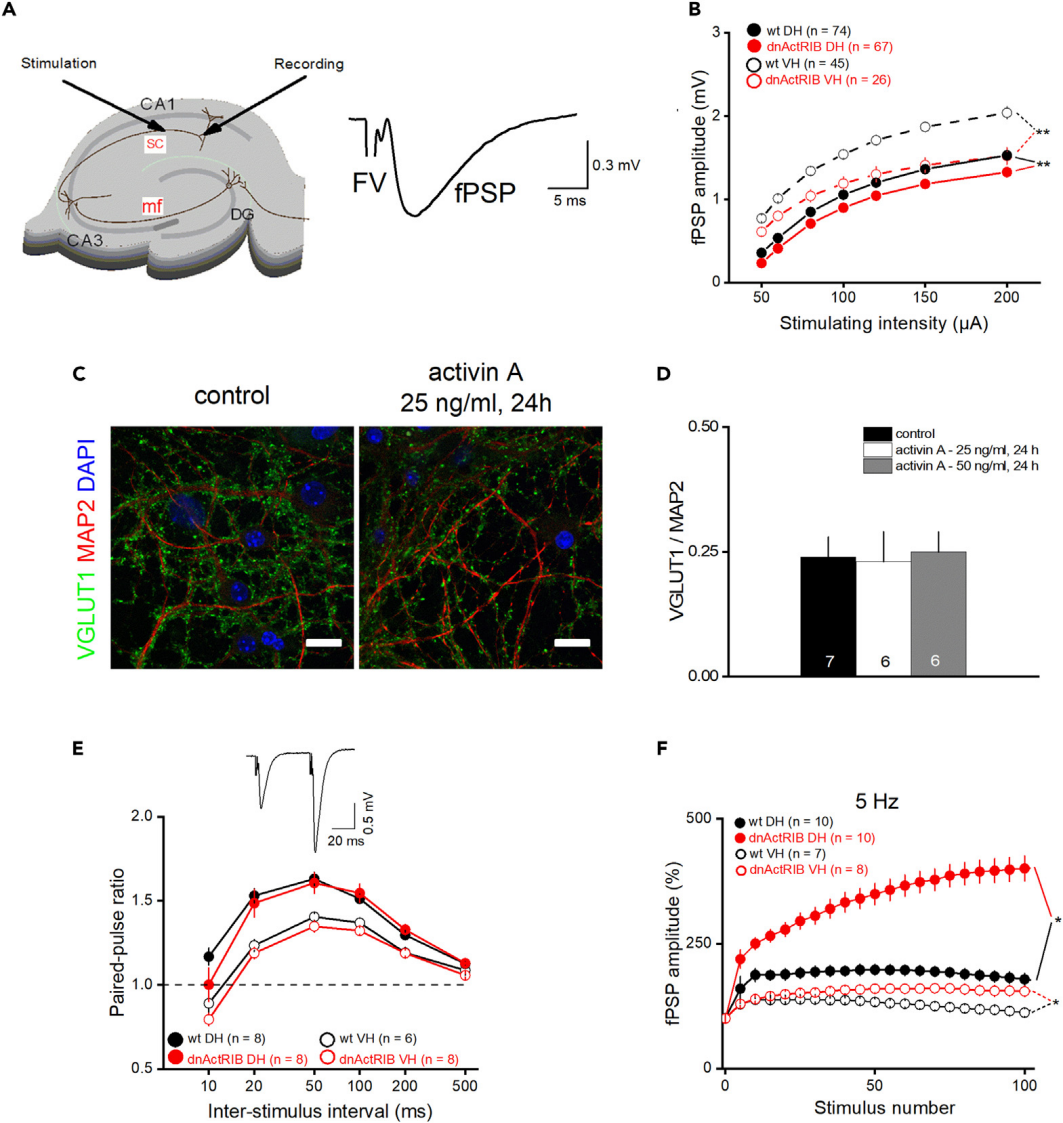
Disruption of activin receptor signaling reduces basal synaptic transmission throughout hippocampus, but strongly augments dorsal short-term facilitation at 5 Hz (A) Schematic illustration of hippocampal slice, indicating sites of stimulation and recording in CA1 stratum radiatum. The illustration on the right side shows a representative field potential trace, consisting of stimulus artifact (truncated), fiber volley (FV), and postsynaptic potential (fPSP). (B) Input-output curves, where fPSP peak amplitude is plotted against stimulus intensity in dorsal CA1 (closed circles) and ventral CA1 (open circles) from wt (black) and mutant mice (red). (C and D) Hippocampal cultures (DIV 20–25) were stained for VGLUT1 (green), MAP2 (red) and DAPI (blue) to visualize synaptic vesicle transporter expression on identified neuronal neurites (C) with or without recombinant activin A (25 ng/mL, 24 h). Scale bar, 20 μm. Histogram of VGLUT1/MAP2 ratio (D) shows no significant effect of activin A on VGLUT1 expression. (E) Paired-pulse stimuli were delivered at inter-stimulus intervals (ISI) ranging from 10 ms up to 500 ms in dorsal CA1 (closed circles) and ventral CA1 (open circles). PPRs in both areas were not affected by lack of activin signaling. Inset in E from a wt slice shows representative PPR at 50 ms ISI. (F) Hippocampal slices from wt (black) and dnActRIB (red) mice containing dorsal CA1 (closed circles) or ventral CA1 (open circles) were stimulated with 100 pulses at 5 Hz. Last 10 pulses were averaged and used for statistical comparison. Values are shown as mean ± SEM. Statistical comparisons were performed using an unpaired, two-tailed Student’s *t* test at α = 0.05 (B, E, and F) or a one-way ANOVA followed by Tukey’s post-hoc test (D). ∗p < 0.05, ∗∗p < 0.01.

In addition to its effects on cellular excitability, we and others have established a role of activin A in glutamatergic transmission and plasticity at the Schaffer collateral (SC)—CA1 synapse.^28^^,^^29^^,^^33^^,^^34^ Like the previous work on firing behavior, those studies focused on DH. In view of the pronounced regional imbalance of activin A levels described here, we were curious how elimination of activin signaling would affect excitatory synaptic function and plasticity in ventral CA1 relative to dorsal CA1. To address this issue, we recorded field potentials in CA1 stratum radiatum that were evoked by electrical stimulation of the Schaffer collateral/commissural pathway (Figure 3A), using slices containing dorsal or VH from wt or dnActRIB mice.

**Figure 4:**
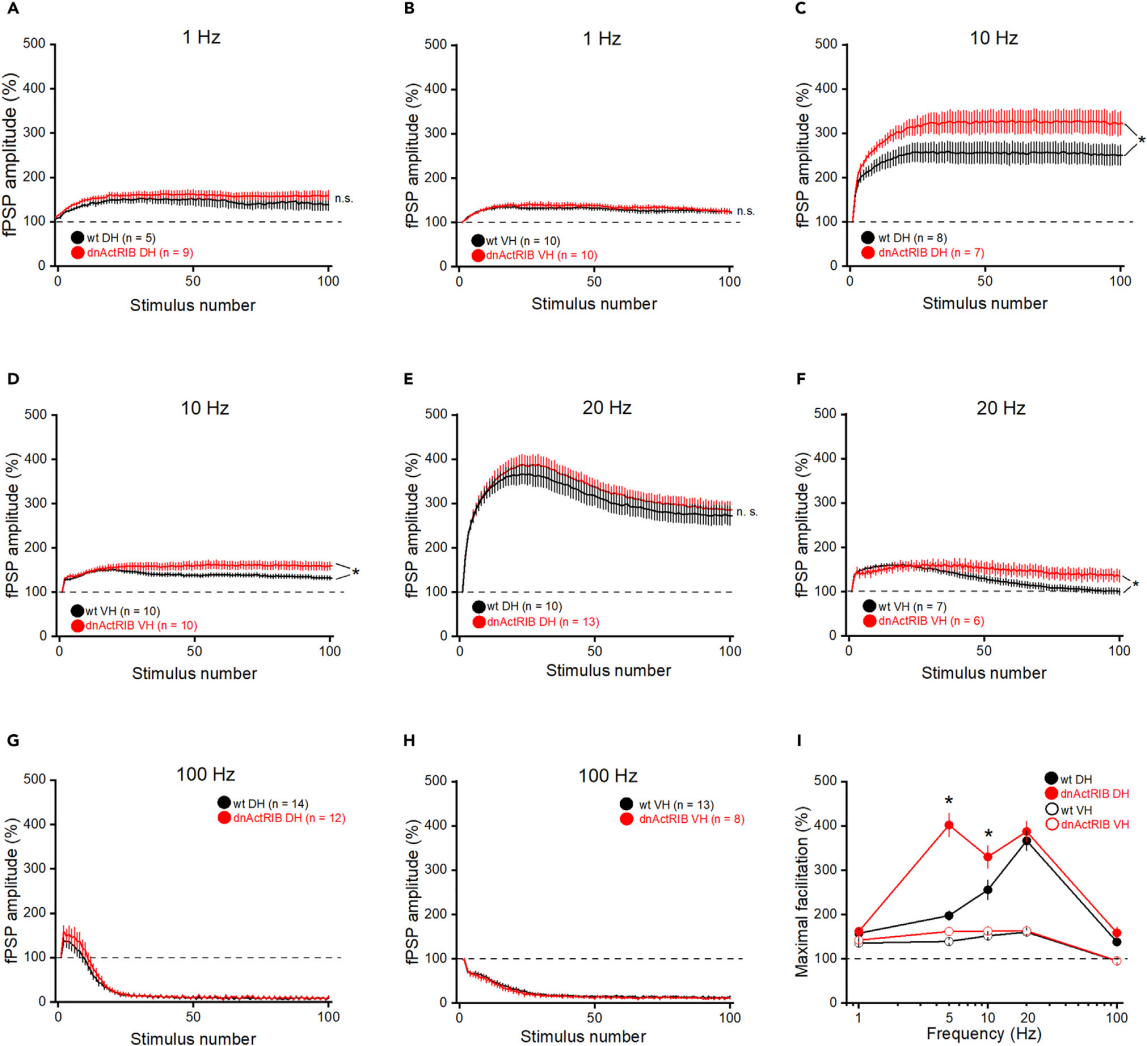
Dorsal CA1 of mutant mice exhibits significantly broadened tuning curve of frequency facilitation (A–H) Hippocampal slices from wt and dnActRIB mice containing dorsal CA1 (A, C, E, and G) or ventral CA1 (B, D, F, and H) were stimulated with 100 pulses at 1 Hz (A and B), 10 Hz (C and D), 20 Hz (E and F), and 100 Hz (G and H). Last 10 responses were used for statistical analysis. (I) Data from A–H were used to construct tuning curves of frequency-dependent short-term plasticity in dorsal and ventral CA1 of both genotypes. Values are shown as mean ± SEM. Statistical comparisons were performed using an unpaired, two-tailed Student’s *t* test at α = 0.05. ∗p < 0.05.

In the first set of experiments, we determined input-output (I-O) curves, which relate peak amplitude of field postsynaptic potentials (fPSPs) to intensity of electrical stimulation. Compared to I-O relationships in slices from wt mice, fPSP responses to increasing stimulus strength were attenuated in DH and, more so, in VH from mutant mice (@ 200 μA; wt DH: 1.53 ± 0.07 mV, n = 74; dnActRIB DH: 1.34 ± 0.04 mV, n = 67; p = 0.005; Figure 4B; wt VH: 2.04 ± 0.07 mV, n = 45; dnActRIB VH: 1.52 ± 0.1 mV, n = 26; p = 6.14e^−5^; Figure 3B). Interestingly, the histone demethylase KDM6B, which we had identified as product of a novel activin target gene,^35^ was recently found to induce the expression of vesicular glutamate transporters VGLUT1/2 in cultured cortical neurons.^36^ It was therefore tempting to speculate that the disruption of activin receptor signaling in our mutant preparation weakens excitatory neurotransmission by impairing the filling of presynaptic vesicles with glutamate. To test the hypothesis that activin has an impact on VGLUT1 expression, we treated cultured hippocampal neurons with recombinant activin A (25 ng/mL or 50 ng/mL) for 24 h and determined the intensity of the VGLUT1 signal in relation to MAP2-stained dendrites. As shown in Figures 3C and 3D, the VGLUT1/MAP2 ratio was not affected by activin A administration (con: 0.24 ± 0.04, n = 7; 25 ng/mL: 0.23 ± 0.06, n = 6, p = 0.970; 50 ng/mL: 0.24 ± 0.04, n = 6, p = 0.950). Thus, VGLUT1 deficiency is unlikely to account for the attenuated fPSP response in our activin signaling-deprived slices. As a side note, this finding does not necessarily argue against an involvement of activin in KDM6B-mediated VGLUT1 expression, as the activity of KDM6B is presumably controlled by several factors in an interdependent or synergistic fashion. In support of this notion, it was found that exposure to an enriched environment engendered rapid up-regulation of *Kdm6b*-mRNA in murine hippocampus at a time point, when *Inhba* mRNA levels were not yet increased.^37^

To assess STP, we calculated paired-pulse ratio (PPR) with stimuli 10–500 ms apart, and a stimulus strength yielding 30% of maximum response (Figure 3E). In line with previous work,^12^ paired-pulse facilitation was stronger in DH than in VH. Loss of activin signaling did not alter shape nor maximum of paired-pulse facilitation in DH and VH (Figure 3E, p > 0.05 for every inter-stimulus interval [ISI]). In striking contrast, the transgene introduced a significant upward shift in fPSP responses to trains of 100 stimuli at 5 Hz, which was particularly prominent in DH (mean facilitation 91^st^ - 100^th^ pulse; wt DH: 169.70 ± 9.60%, n = 7; dnActRIB DH: 368.32 ± 33.33%, n = 7, p = 0.90e^−4^; wt VH: 115.57 ± 7.55%, n = 10; dnActRIB VH: 155.38 ± 10.29%, n = 10, p = 0.008; Figure 3F).

We then systematically varied stimulation frequencies between 1 and 100 Hz. At 1 Hz, frequency-dependent facilitation did not differ between wt and mutant hippocampi, neither in dorsal CA1 (wt: 135.40 ± 9.64%, n = 5; dnActRIB: 146.21 ± 7.58%, n = 9; p = 0.41; Figure 4A), nor in ventral CA1 (wt: 124.62 ± 7.57%, n = 10; dnActRIB: 123.70 ± 6.13%, n = 10; p = 0.63; Figure 4B). At 10 Hz, facilitation in mutant hippocampi was not as pronounced as at 5 Hz, but still significant in both DH (wt: 251.40 ± 23.00%, n = 8; dnActRIB: 341.55 ± 23.35%, n = 7; p = 0.019, Figure 4C) and VH (wt: 133.64 ± 5.60%, n = 10; dnActRIB VH: 159.51 ± 8.21%, n = 10; p = 0.020; Figure 4D). At 20 Hz, facilitation in wt dorsal CA1 attained its maximum, which was not further increased in the mutant counterpart, possibly reflecting a ceiling effect. In ventral CA1, loss of activin signaling stabilized the weak and transient augmentation seen in wt (DH: wt 273.10 ± 26.62%, n = 10; dnActRIB: 309.67 ± 27.13%, n = 13; p = 0.33; Figure 4E, VH wt: 101.67 ± 5.65%, n = 7; dnActRIB VH: 137.37 ± 12.52%, n = 6; p = 0.023; Figure 4F). Stimulation at 100 Hz led to a rapid and equal decline of synaptic responses in all four groups, preceded only by brief facilitation in DH of either genotype (Figures 4G and 4H).

From the graphic summary, which plots maximum facilitation as function of stimulation frequency, genotype, and hippocampal region (Figure 4I), it becomes evident that facilitation is markedly stronger in dorsal than ventral CA1. Furthermore, in dorsal CA1, loss of activin signaling entails a massive broadening of the frequency band where fPSPs receive the strongest enhancement. Whereas dorsal CA1 in wt hippocampus exhibits a rather confined frequency range of maximum facilitation with an apparent peak around 20 Hz, this window is extended to much lower frequencies (5–10 Hz) in the mutant preparation, thereby blurring the characteristic frequency selectivity of the wild type.

### Activin signaling affects long-term synaptic plasticity in dorsal, but not ventral CA1

**Figure 5:**
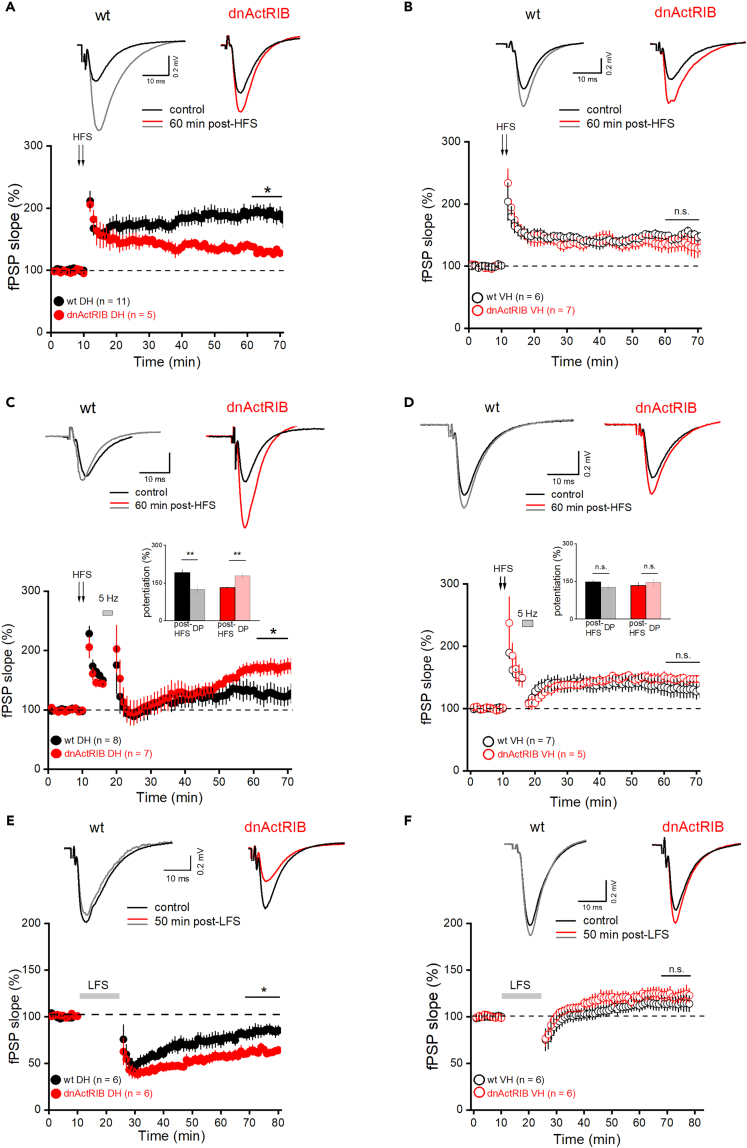
Elimination of activin signaling impacts long-term synaptic plasticity only in dorsal CA1 (A) CA1 HFS-LTP was reduced in dorsal dnActRIB slices compared to their wt counterparts. (B) HFS-LTP in ventral CA1 was indistinguishable between wt and dnActRIB slices. Insets depict field potentials before (black traces) and 60 min after HFS (wt: gray; dnActRIB: red) in dorsal CA1 (A) and ventral CA1 (B). (C and D) De-potentiation protocol consisting of 5 Hz stimulation (for 3 min) 5 min after 2x HFS in dorsal (C) and ventral (D) wt and dnActRIB slices. Traces above depict field potentials before (black traces) and 50 min after HFS (wt: gray; dnActRIB: red) in dorsal CA1 (C) and ventral CA1 (D). Bar charts in the center of the panels compare extent of fPSP change 51–60 min post-HFS without (from A and B, labeled “post-HFS”) and with 5 Hz stimulation (from C and D, labeled “DP”). (E and F) Paired-pulse low-frequency stimulation (PP-LFS, 1 Hz for 15 min, 50 ms ISI) was used to induce LTD. Note enhanced LTD in dorsal dnActRIB slices (E), whereas PP-LFS failed to induce LTD in ventral slices of both genotypes (F). Insets depict representative voltage traces before and 50 min after LFS. Values are shown as mean ± SEM. Statistical comparisons were performed using an unpaired, two-tailed Student’s *t* test at α = 0.05. ∗p < 0.05, ∗∗p < 0.01.

We next asked whether elimination of endogenous activin signaling would alter long-term plasticity with the same dorsal bias as observed for STP. For induction of robust LTP, we used a standard high frequency stimulation (HFS) protocol (2 × 100 Hz for 1 s, 20 s apart).^38^ LTP in wt dorsal CA1 (51–60 min post-HFS: 191.39 ± 11.36%, n = 11) was strongly decreased in mutant slices (130.87 ± 2.24%, n = 5; p = 0.003; Figure 5A), whereas for ventral CA1, we did not observe an appreciable difference between LTP in wt (147.10 ± 5.14%, n = 6) and mutant (133.65 ± 13.22%, n = 6; p = 0.370; Figure 5B). Consistent with the majority of previous studies comparing LTP between DH and VH,^9^ the magnitude of LTP was significantly larger in dorsal than in ventral CA1 of wt hippocampus (p = 0.014).

We next investigated the effect of endogenous activin signaling on immediate de-potentiation (DP) of LTP. DP was achieved by means of a 3 min stimulus train at 5 Hz delivered 5 min after HFS.^38^ In wt DH slices, DP was successful in that the intervention engendered a strong reduction of potentiation compared to HFS alone (wt HFS +5 Hz: 124.41 ± 16.61%, n = 7, p = 0.003 vs. HFS alone, Figure 5C). In mutant DH, however, the 5 Hz regimen failed at de-potentiating LTP to such an extent that fPSPs grew even larger than with HFS alone (HFS +5Hz: 177.73 ± 12.52%, n = 7; p = 0.008 vs. HFS alone; Figure 5C). Intriguingly, DP did not work in ventral CA1, independent of genotype (wt-VH HFS +5 Hz: 126.86 ± 9.17%, n = 7; HFS alone: 147.10 ± 2.64%, n = 6; p = 0.120; mutant-VH HFS +5 Hz: 145.74 ± 13.18%, n = 5; HFS alone 133.65 ± 5.04%, n = 6, p = 0.440; Figure 5D).

Finally, we examined whether LTD would be also modulated by activin in a site-specific fashion. LTD was induced by low-frequency stimulation (LFS, 1 Hz) with paired stimuli (50 ms apart) for 15 min. In dorsal CA1, the magnitude of LTD obtained in wt slices (51–60 min post-LFS; 85.67 ± 5.28%, n = 6; Figure 5E) was significantly enhanced in mutant slices (51–60 min post-LFS: 62.08 ± 3.51% of baseline, n = 6; p = 0.004, Figure 5E). This finding is in line with our earlier observation that application of recombinant activin A has the opposite effect, namely a significant decrease in LTD.^33^ Notably, the very same LFS protocol that reliably induced robust LTD in dorsal CA1, failed to do so in the ventral analogue. Instead, in both wt and mutant VH slices, synaptic responses rapidly recovered after LFS and remained even slightly enhanced above control levels (51–60 min post-LFS; wt VH: 114.54 ± 8.04%, n = 5; mutant VH: 122.06 ± 6.27%, n = 5; p = 0.48; Figure 5F). Taken together, our findings strongly suggest that endogenous activin signaling serves to augment LTP, to ensure its rapid de-potentiation, and to dampen LTD, with all these effects restricted to dorsal CA1.

**Figure 6:**
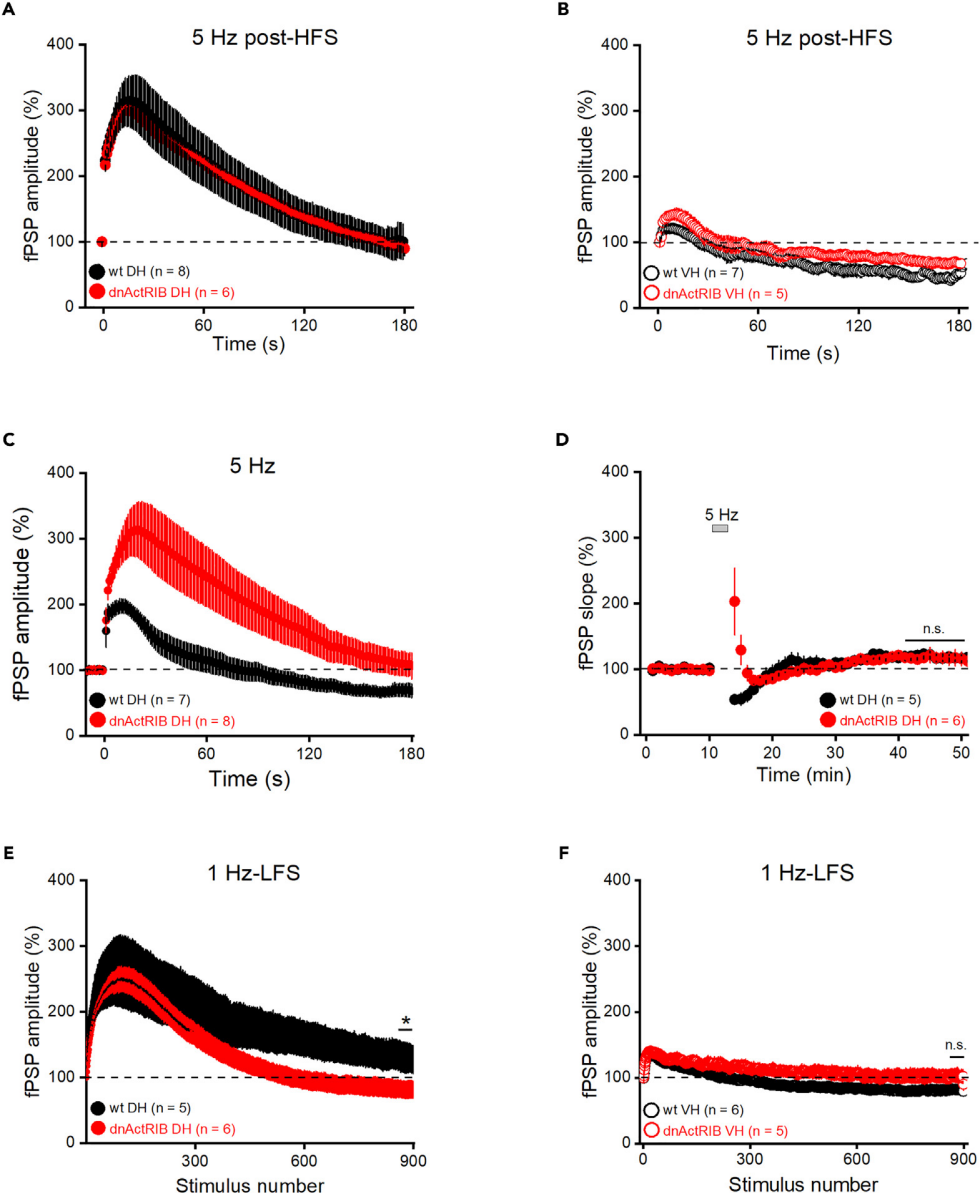
Analysis of fPSP responses during induction protocols for de-potentiation and LTD (A, C, and D) fPSP trajectories in dorsal CA1 from wt and mutant mice are virtually identical during the 3 min period of 5 Hz stimulation when determined 5 min after HFS (A). Without preceding HFS, however, the same 3-min-5-Hz paradigm yields strikingly divergent trajectories (C), as expected from the strong difference between the genotypes in 5 Hz short-term plasticity (cf. Figure 4G). The strikingly augmented 5 Hz facilitation in the absence of activin signaling (red trace in C) remains a transient phenomenon without distinct effect on fPSP slope in the long run (D). (B, E, and F) The remaining panels show fPSP behavior during de-potentiation induction in ventral CA1 (B), and during PP-LFS in dorsal CA1 (E) and in ventral CA1 (F) for both genotypes. Values are shown as mean ± SEM. Statistical comparisons were performed using an unpaired, two-tailed Student’s *t* test at α = 0.05. ∗p < 0.05.

So far, we had always focused on the outcome of the various plasticity-inducing protocols at the end of the recording session. We next turned to the behavior of synaptic responses during stimulation, as discrepancies within this period might possibly account for distinct long-term effects. For example, we speculated that the marked increase of frequency-dependent facilitation at 5 Hz in mutant DH reported earlier (Figure 3F) could offer a likely explanation for the abortive de-potentiation in the dorsal mutant (Figure 5C). Therefore, we monitored fPSP responses during the entire 3 min, 5 Hz de-potentiation protocol 5 min after HFS or, as a control, without preceding HFS. In the latter experiment, naive slices from mutant DH showed the hugely augmented synaptic responses (Figure 6C), as expected from the aberrant behavior at STP (Figure 3F). On a side note, the striking discrepancy in the response size during the 3 min of 5 Hz stimulation did not entail different long-term effects between the two genotypes. In both groups, we observed a slight, lasting potentiation (41–50 min post 5 Hz stimulation, wt: 119.26 ± 5.38%, n = 5; mutant: 117.96 ± 7.27%, n = 6; p = 0.88; Figure 6D). When the response pattern during the DP-inducing protocol was re-examined 5 min post HFS, we obtained an almost perfect overlap of wt and mutant DH (Figure 6A). Whereas the preceding tetanization did not produce a further increase in mutant DH, the corresponding fPSPs in wt slices were now elevated to such a level that their trajectory almost perfectly matched that obtained in the mutant preparation. Maximum facilitation amounted to 327.21 ± 37.16% in wt slices (n = 8) and to 308.57 ± 24.80% in mutant slices (n = 6; p = 0.71; Figure 6A). This finding does not lend support to our initial hypothesis that a deviant response during induction of de-potentiation might explain its failure in mutant DH slices.

From a comparison of Figures 6A and 6B, it seems, however, plausible to link the absent de-potentiation in ventral CA1 of either genotype to the almost entirely missing response facilitation during the induction protocol. Compared to the 300% plus boost in wt dorsal CA1 detailed earlier, fPSPs of wt ventral CA1 rose only to a meager and short-lived potentiation (129.81 ± 5.57%, n = 6, p = 0.0003; Figures 6A and 6B).

We next analyzed the synaptic responses recorded during the LTD induction protocol, based on the amplitude of the first of the fPSP twins evoked by our paired-pulse LFS protocol (PP-LFS). Again, facilitation was much stronger in dorsal than ventral CA1 of wt slices (maximal facilitation around the 91^st^–100^th^ pulse, wt DH: 246.53 ± 53.44%, n = 5; wt VH: 118.91 ± 10.77%, n = 6; p = 0.016; Figure 6E). Note that, compared to single-pulse stimulation at 1 Hz (Figure 4A), delivery of paired pulses at the same frequency made a huge difference in terms of fPSP amplification during LFS. This discrepancy offers a plausible explanation of why the paired-pulse variant of LFS is essential to induce LTD in dorsal CA1. In both regions, the initial facilitation decayed over time, with dorsal responses remaining above baseline until the end of LFS (last ten stimuli, 121.82 ± 19.68%, Figure 6E), whereas the ventral trajectory fell below baseline (last ten stimuli, 80.93 ± 11.33%, Figure 6F), as also observed during the ventral DP-inducing protocol (Figure 6B).

In dorsal CA1, response trajectories during PP-LFS attained initially an overlapping maximum in both genotypes. Possibly portending stronger LTD, field responses in the mutant declined faster and fell below baseline compared to those in wt, which stayed above baseline throughout LFS sessions (last ten stimuli, wt DH: 121.82 ± 19.68%, n = 5; dnActRIB: 72.59 ± 10.54%, n = 6; p = 0.046; Figure 6E). During delivery of PP-LFS, PPR decreased initially, but then remained stable at around 75% of control, irrespective of genotype and hippocampal region (ratio of averaged last 20 PPRs to first PPR for dorsal CA1, wt: 0.62 ± 0.06, n = 5; dnActRIB: 0.72 ± 0.05, n = 6, p = 0.3; and for ventral CA1, wt: 0.78 ± 0.03, n = 6; dnActRIB: 0.79 ± 0.02, n = 5, p = 0.73).

### LTD induction is associated with decreased fiber excitability, independent of activin signaling

LTD at the SC-CA1 synapse is commonly attributed to a postsynaptic mechanism.^39^ Here, we made the unexpected observation that the fiber volley (FV), which precedes fPSPs and reflects stimulus-evoked AP discharge in the Schaffer pathway, did not remain stable during LFS and subsequent LTD, pointing to a presynaptic site of LTD (*data not shown*). To interrogate the role of axonal excitability in dorsal LTD in more detail, we performed a series of experiments, in which the ionotropic glutamate receptor antagonist kynurenic acid (2 mM) and the GABA_A_ receptor antagonist picrotoxin (100 μM) served to pharmacologically isolate FVs.

**Figure 7:**
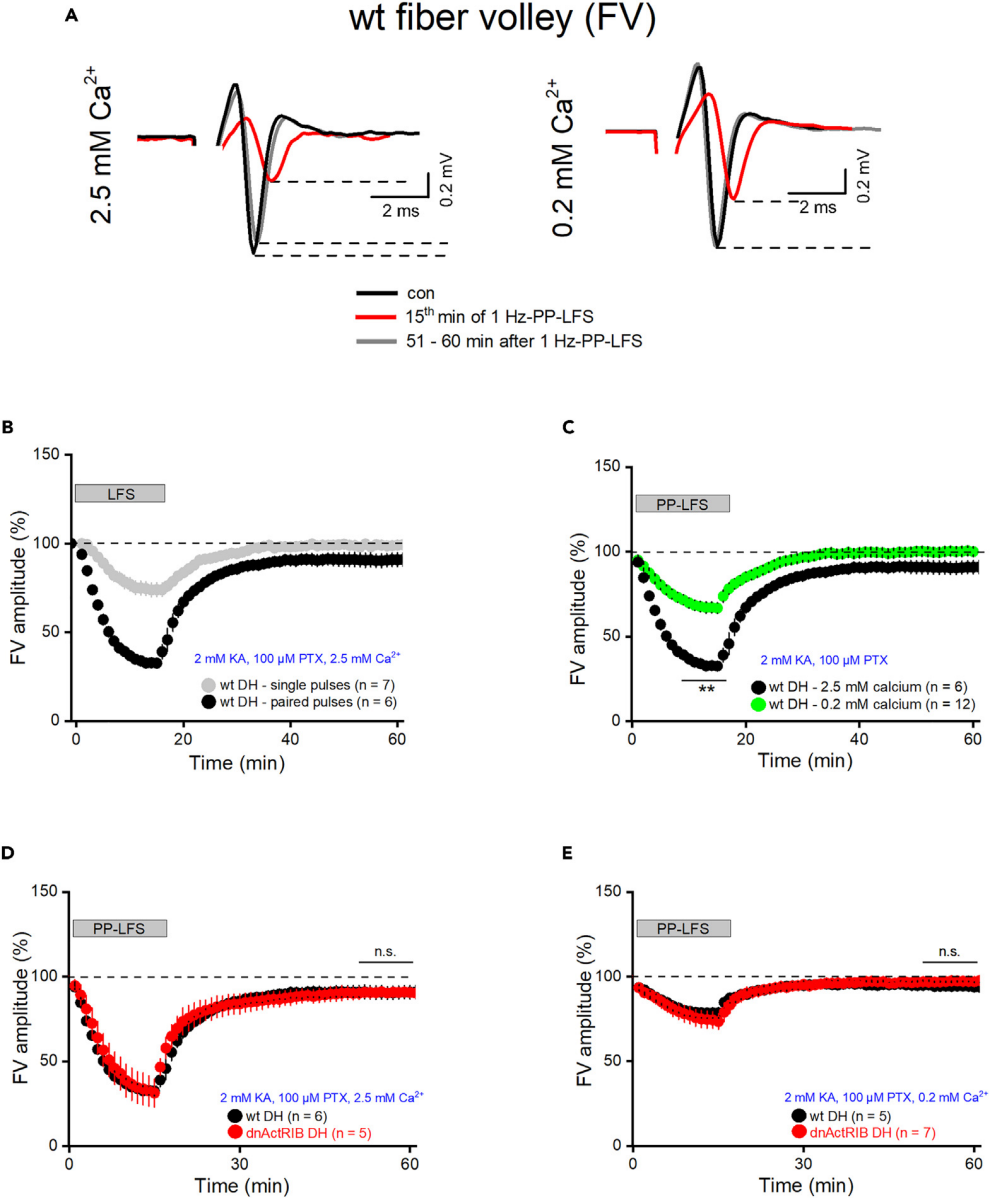
LTD induction in dorsal CA1 coincides with transient fiber volley (FV) suppression, which does not fully recover during LTD expression FVs were recorded after pharmacologic inhibition of fast glutamatergic and GABAergic transmission. (A) Superimposition of FV traces before, at the end of 15 min PP-LFS, and about 1 h after PP-LFS, in normal extracellular Ca^2+^ (left) and in low extracellular Ca^2+^ (right). (B) Differential effect of single vs. paired-pulse LFS on FV amplitude. (C) FV suppression by PP-LFS is strongly mitigated in low Ca^2+^. (D and E) Disruption of endogenous activin signaling did not affect FV changes during and after LFS, neither in normal (D) nor in low (E) Ca^2+^. Values are shown as mean ± SEM. Statistical comparisons were performed using an unpaired, two-tailed Student’s *t* test at α = 0.05. ∗∗p < 0.01.

When subjected to our standard PP-LFS protocol, FV amplitudes in wt dorsal CA1 plummeted to roughly one-third of control (last 5 min of LFS: 33.43 ± 2.45%, n = 6) and did not show full recovery thereafter, as the mean amplitude 35–40 min post LFS amounted to only 90.73 ± 3.31% of control (n = 6, Figures 7A and 7C). Lending credence to the notion that presynaptic excitability is involved in LTD, we found that single-pulse LTD (SP-LFS), which normally fails to induce LTD in adult animals, entails much weaker reduction of FV amplitude (last 5 min of LFS; 77.27 ± 3.90%, n = 7; p = 3.60e^−6^ vs. PP-LFS), which also completely recovered to baseline when determined 35–40 min post LFS (98.97 ± 1.82%, n = 6; Figure 7B).

The impact of PP-LFS on FV amplitude depended strongly on extracellular Ca^2+^ concentration. When we lowered Ca^2+^ in the bathing solution from 2.5 to 0.2 mM (with compensatory increase of Mg^2+^ from 1.5 to 3.8 mM), FV depression during PP-LFS was strongly mitigated, as amplitude declined to only 67.39 ± 3.18% of control (n = 13), and full recovery to 99.85 ± 1.80% of control (n = 11) was obtained within the recording session (Figures 7A and 7C).

Given that wt and mutant CA1 pyramidal cells exhibited some differences regarding somatically evoked AP waveforms (Table 2), we asked next whether endogenous activin signaling would affect FV responses during and after PP-HFS. However, for both normal and low extracellular Ca^2+^ conditions, FV trajectories from mutant CA1 were virtually identical to those from the wt analogue (Figures 7D and 7E). Thus, it appears unlikely that the enhanced LTD in area CA1 of mutant mice has a presynaptic origin.

## Discussion

In our study, we used a transgenic mouse line with disrupted activin receptor signaling to establish a close relationship between the strong dorsal-ventral gradient of activin A protein in the hippocampus and the asymmetric distribution of electrophysiological properties that govern cellular excitability and synaptic plasticity in dorsal and ventral CA1. Consistent with previous studies,^4^^,^^5^^,^^6^^,^^9^ ventral CA1 pyramidal cells from wild-type hippocampus displayed a significantly higher firing propensity upon ramp depolarization than their dorsal counterparts, which we attribute to less negative RMP and higher R_N_ in the former. Elimination of endogenous activin signaling removed the dorsal-ventral difference in RMP and increased dorsal R_N_, which resulted in a region-specific increase in excitability. With their RMPs aligned and R_N_ increased dorsally, the firing response to ramp depolarization became indistinguishable between mutant CA1 neurons from the two regions. Interestingly, in a study on neuromuscular junction in *drosophila melanogaster,* elimination of the activin-specific type 1 receptor *babo* or its downstream signaling was found to depolarize RMP of the muscle cell, accompanied by reduced mRNA level of Na^+^-K^+^-ATPase α-subunit.^40^ In that study, genetic interference with activin signaling led also to altered mRNA transcripts of a number of K^+^ channels.^40^ This finding is of interest, too, as the striking surge in activin A that is caused by the exploration of an enriched environment promotes firing of dentate gyrus granule cells through suppression of G protein-activated inwardly rectifying K^+^ (GIRK) current.^32^ Thus, it seems conceivable that the basal (or tonic) level of activin A, which apparently dampens firing (as inferred from our recordings from dnActRIB neurons), might produce an effect on cellular excitability that is inverse to that following a phasic rise of activin A, which strengthens firing. It remains to be determined whether such opposing, concentration-dependent effects of activin A are segregated with respect to the signaling pathway involved (canonical, Smad-dependent signaling vs. non-canonical signaling including PKC, MAPK, and others).

Since expression of the mutant activin receptor (dnActRIB) was driven by the CaMKII-α promoter, the transgene should not have appeared before late postnatal development, with its expression being restricted to principal (glutamatergic) neurons in the forebrain. Nevertheless, the question remains whether the electrophysiological effects observed in adult dnActRIB neurons are a direct consequence of the lack of activin receptor signaling or rather reflects adaptive changes over time. In a previous study on (glutamatergic) mossy cells in the hilus of the dentate gyrus,^41^ we found that administration of recombinant activin A to wild-type neurons produced electrophysiological effects exactly opposite to those observed in dnActRIB neurons. It is therefore plausible to assume that the altered excitability of mutant mossy cells and, by analogy, of the mutant CA1 pyramidal cells examined here, arose directly from disrupted activin receptor signaling. Of note, hilar interneurons, which are not supposed to express the transgene, also showed changes in their firing properties when measured in slices from dnActRIB mice.^41^ Thus, elimination of endogenous activin signaling in one cell type may entail adaptive changes in other neurons of the hippocampal network.

At the Schaffer collateral—CA1 pyramidal cell synapse, dnActRIB slices showed a significantly lowered I-O relationship. Whereas the dorsal > ventral distribution of paired-pulse facilitation was not affected in mutant slices, stimulation frequencies of 1–100 Hz, with 100 pulses each, revealed deviant use-dependent plasticity, mainly in dorsal CA1. Aberrant behavior was particularly striking at low theta frequency (5 Hz): In wt dorsal CA1, fPSPs attained a stable plateau of facilitation within the first 10 stimuli. In sharp contrast, fPSPs from mutant dorsal CA1 exhibited biphasic augmentation, with a steep initial rise in amplitude followed by a flatter, but continuously growing trajectory. In functional terms, the dramatically augmented response to theta stimulation in mutant dorsal CA1, together with the concomitant rise in postsynaptic excitability, is likely to impair information transfer, as presynaptic facilitation would drive the postsynaptic neuron soon to fire at its maximum rate, thereby causing loss of information. Thus, activin signaling might serve as a means to match presynaptic facilitation to postsynaptic excitability in order to optimize coding.

Regarding the underpinnings of use-dependent facilitation, various mechanisms have been proposed including, among others, the modified residual Ca^2+^ hypothesis with a second (high-affinity, slow kinetics) Ca^2+^ sensor for facilitation, Ca^2+^ buffer saturation, broadening of presynaptic spike, and ratio of fast to slow vesicles pools.^42^ While the targets of activin signaling that control augmentation remain to be elucidated, the biphasic nature of augmentation in mutant dorsal CA1 suggests that two different mechanisms should be involved.

So far, we have described the massively enhanced facilitation at 5 Hz as aberrant behavior, but we might have to re-consider this classification in light of the observation that exactly this augmentation is seen in wt dorsal CA1 when a 5 Hz stimulus train is delivered 5 min after HFS. This phenomenon, which we tentatively call “post-tetanic potentiation of frequency-dependent facilitation” raises the question of whether activin signaling normally puts a brake on the underlying mechanism(s) that is only released during HFS. In view of the apparent disinhibition of facilitation at 5 Hz that we see in the mutant already before HFS, one would expect stronger glutamate release in mutant than in wild-type CA1, if, instead of HFS, more physiological theta bursts stimulation (TBS) is used to induce LTP. We have previously shown, however, that, despite the presumed stronger presynaptic drive, TBS-induced LTP is significantly weaker in mutant CA1.^28^ This apparent paradox suggests that the postsynaptic deficit in dnActRIB neurons, manifested in a reduced NMDA current component, cannot be overridden by a (compensatory?) enhancement in glutamate release during TBS.

Does NMDA receptor hypofunction also account for the failed depotentiation in our mutant preparation? Previous work showed that the reversal of LTP depends on NMDA receptor activation.^43^^,^^44^ Thus, the decrease in NMDA receptor activity that we have observed in dnActRIB CA1 neurons^28^ might have indeed contributed to the lack of depotentiation. It is worth noting, however, that a number of additional players have been implicated in depotentiation, including protein kinases and phosphatases, cAMP, Ca^2+^ release from intracellular stores, and adenosine receptors.^45^ Whereas several of the pathways involved in depotentiation are at least partially overlapping with signaling cascades of LTP and LTD, calcineurin (protein phosphatase 2B) is a remarkable exception. Genetic ablation of its Aα isoform selectively abolished depotentiation, but affected neither LTP nor LTD.^38^ Although it is conceivable that activin A targets calcineurin Aα function to suppress depotentiation, additional effects must exist to explain the factor’s broad spectrum of actions on synaptic plasticity. It will be a demanding project for future studies to elucidate the molecular pathways through which activin receptor signaling provides a well-balanced relationship between synaptic potentiation and depression.

We report here that the dorsal-ventral difference in HFS-LTP magnitude in wt hippocampus is abrogated in dnActRIB hippocampus. In a previous study, the dorsal-ventral LTP gradient was related to differences in the expression pattern of plasticity-associated neurotransmitter receptors, with the surprising finding that protein levels of the NMDA receptor subunits GluN1 and GluN2B were significantly higher in ventral than in dorsal CA1 stratum radiatum.^9^ Our data offer a resolution to this apparent paradox. Activin receptor signaling has been shown to augment NMDA receptor currents in hippocampal neurons.^28^^,^^46^ Thus, the prominent dorsal-ventral activin A gradient should be poised to more than compensate for the lower NMDA receptor level in dorsal CA1, thereby conferring higher LTP functionality upon this hippocampal region. In addition, activin signaling might control the local strength of LTP through a region-specific modulation of tonic inhibition mediated by extrasynaptic GABA_A_ receptors. We have previously shown that basal activin receptor signaling dampens the GABA tone in wt dorsal CA1 pyramidal cells, which should facilitate LTP induction in DH.^30^ By contrast, tonic GABA inhibition is significantly stronger in wt ventral CA1 pyramidal cells and, as expected from the low basal level of endogenous activin A in VH, remains unchanged in ventral CA1 neurons from dnActRIB mice.^47^ Thus, the ventral-to-dorsal gradient of the GABA tone that is established by the inverse gradient of activin A should be an important mechanism to bias LTP in favor of DH. In strong support of this notion, Pofantis and Papatheodoropoulos (2014) reported that pharmacological blockade of α5-containing GABA_A_ receptors,^48^ which are, due to their extrasynaptic location, the main constituent of the GABA tone in CA1,^49^ abolished the difference in LTP between dorsal and ventral CA1.

Since standard single-pulse LFS (1 Hz for 15 min) becomes less effective at inducing LTD as the hippocampus matures, a number of modified protocols have been employed to elicit LTD in the adult preparation, including paired-pulse LFS (PP-LFS) and low-frequency burst stimulation (LFBS).^50^ With respect to LTD expression along the longitudinal axis of the hippocampus, two studies reported no dorsal-ventral differences. In slices from juvenile rats, PP-LFS induced LTD of virtually identical size in dorsal and ventral CA1.^51^ Equivalent LTD in dorsal and ventral CA1, this time in response to LFBS, was also observed in slices from young rats (6–10 weeks).^9^ Contrary to these studies in rat hippocampus, we found that PP-LFS induced LTD only in dorsal CA1, with the extent of LTD being doubled in the mutant slice. In ventral CA1 from both genotypes, however, PP-LFS failed to elicit LTD. Thus, in contrast to dorsal CA1, where the basal level of activin A is much higher than ventrally, elimination of tonic activin receptor signaling did not affect LTP, de-potentiation and LTD in ventral CA1. However, given that the main signal-transducing receptor ActRIB is equally expressed throughout the hippocampus (Figure 2B), this is not to say that the VH will prove unresponsive to a strong *phasic* rise in activin A, as seen in this sub-region after exposure to an enriched environment.^47^ The functional impact of modulating LTP and LTD in VH was already demonstrated for the dopaminergic system.^52^ It will be interesting to determine in future studies whether activin A, while not tonically framing the window of long-term plasticity as it does in DH, assumes a role in activity-induced remodeling of neuronal circuits in VH.

Intriguingly, induction of LTD in dorsal CA1 was accompanied by a substantial drop in FV amplitude during delivery of PP-LFS. Moreover, fiber volleys did not fully recover after PP-LFS, but remained below baseline for the rest of the recording session, suggesting that reduced axonal excitability should make a presynaptic contribution to the expression of LTD. Contrary to PP-LFS, single-pulse LFS, which did not induce LTD in our hands (*data not shown*), engendered a much weaker and fully reversible attenuation of fiber volleys.

Regarding the fidelity of axonal responses to increasing stimulation frequencies, we and others have previously shown that it takes stimulus trains with frequencies > 20 Hz to curtail axonal excitability.^53^^,^^54^^,^^55^ Why then would a 1 Hz stimulation paradigm produce such a pronounced suppression of FV responses? At such low frequency, accumulation of extracellular K^+^ that drives Na^+^ channels toward inactivation—a common explanation for axonal failure during HFS—seems an unlikely scenario. To solve the apparent paradox that Schaffer collaterals follow 10 Hz unabatedly, but wane at 1 Hz, one should remember the highly different time windows over which axonal behavior is studied, comprising seconds for HFS, but 15 min for LFS. Thus, a mechanism might account for LFS-associated FV depression that kicks in too slowly to affect a 10 Hz stimulus train.

Interestingly, PP-LFS produced much less inhibition when delivered in a low Ca^2+^/high Mg^2+^ bath solution. Surface screening effects offer an unlikely explanation, because then basal excitability of Schaffer collaterals should be altered, too, which is not the case.^56^ A novel and unorthodox way of thinking about this issue comes from a recent study showing that Ca^2+^-evoked release of the glio-transmitter ATP from astrocytes regulates axon excitability and conduction in an activity-dependent fashion.^57^ Adopting this concept, which was developed for myelinated axons from cortical pyramidal cells, to distal Schaffer collaterals, which are unmyelinated,^58^ the following sequence of events might occur: Sustained 1 Hz delivery of paired stimuli, but not of single stimuli, is sufficient to raise intracellular Ca^2+^ in nearby astrocytes, leading to the release of ATP, which is then converted to adenosine, which eventually dampens axonal excitability through intermediate steps. In low Ca^2+^ solution, however, astrocytic Ca^2+^ stores would be gradually depleted, resulting in a decline of ATP release and, consequently, much less inhibition of axonal fibers.

The next question relates to the origin of the small, but sustained depression of fiber volleys that coincides with LTD in fPSP recordings. Does it result from a long-lasting effect of the gliotransmitter, or is it caused by an independent mechanism? An argument for the latter comes from an imaging study on morphological changes of presynaptic boutons during hippocampal LTD.^59^ Induction of LTD entailed a massively enhanced turnover of boutons, leading to substantial remodeling of synaptic circuits. It seems plausible to assume that the morphological restructuring at the presynaptic site would also affect the geometry of axonal branch points. Since these are critical sites for conduction failures,^60^ subtle changes might sum up to cause a small, but significant reduction of FV amplitude, as observed in our recordings.

Whereas the molecular underpinnings of the surprising behavior of fiber volleys during and after LTD induction await further studies, our data allow the following conclusions: Firstly, they lend support to the notion that LTD at the Schaffer-CA1 synapse has an appreciable presynaptic component. Secondly, because elimination of activin receptor signaling did not affect any of the FV recordings, the effects of activin A on LTD should be postsynaptic in nature.

### Limitations of the study

The limitations of our study are inherent in the limitations of the brain slice preparations. Whereas the neuronal components of the local networks and their connectivity are well preserved in the hippocampal slice and hence accessible to detailed interrogation, afferent, and efferent pathways to and from the hippocampus are severed, including the projections from neuromodulatory nuclei that regulate hippocampal function in a behavioral state-dependent fashion. Thus, causal links between electrophysiological features in the slice and behavioral phenotypes are notoriously difficult to establish, and we refrained here largely from doing so. With our study focusing on the impact of tonic activin signaling on firing properties and synaptic plasticity of CA1 pyramidal cells, we delineated a set of electrophysiological patterns that are governed by the factor’s grossly different basic activity in DH vs. VH. This does not imply, however, that the characteristic profile of intrinsic and synaptic properties that we attribute to tonic activin signaling might not be subject to considerable change as a living animal and, even more, a human being, has to interact with the vagaries of a real-life environment.

## STAR★Methods

### Key resources table

**Table:** 

REAGENT or RESOURCE	SOURCE	IDENTIFIER
**Antibodies**		

Mouse anti-β-actin-peroxidase	Sigma-Aldrich	Cat#A3854; RRID: AB_262011
Rabbit anti-activin receptor IB/ALK4	Abcam	Cat#ab109300, antibody registry AB_10860328
Goat anti-rabbit IgG H&L (HRP)	Abcam	Cat#ab6721, RRID: AB_955447
Rabbit monoclonal anti-VGLUT1	Synaptic Systems	Cat#135308, antibody registry: AB_2864787
Guinea pig anti-MAP2	Synaptic Systems	Cat#188004, RRID: AB_2138181
Donkey-anti-guinea pig Cy3	Thermo Fisher Scientific	Cat#A21206, RRID: AB_2535792
Donkey-anti-rabbit Alexa488	Thermo Fisher Scientific	Cat# A-21206, RRID: AB_2535792

**Chemicals, peptides, and recombinant proteins**		

Picrotoxin	Sigma-Aldrich	CAS 124-87-8, cat. # 528105
Kynurenic acid	Sigma-Aldrich	CAS 492-27-3, Cat#K3375
Recombinant Activin A	Bio-techne/RD systems	Cat#338AC

**Critical commercial assays**		

Quantikine Activin A ELISA kit	Bio-techne/RD systems	Cat#DAC00B

**Experimental models: Organisms/strains**		

Mouse: C57Bl/6J (JAX® Mice Strain)	Charles River	Strain code: 632
Mouse: dnActRIB transgenic mouse line	Müller et al.^28^	N/A
Mouse primary hippocampal culture derived from C57Bl/6J	Charles River	Strain code: 632

**Oligonucleotides**		

dnActRIB fw: 5’-tctaccataaccgccagagg-3’	Eurofins	NM_007395.3
dnActRIB rev: 5’-aggtcctcctcggaaatcag-3’	Eurofins	NM_007395.3
ActRIB fw: 5’-gtggggaccaaacgatacat-3’	Eurofins	N/A
ActRIB,rev: 5’-tcatggactcctccagaattg-3’	Eurofins	N/A
TBP fw: 5’-gccaagagtgaagaacaatcc-3’	Eurofins	N/A
TBP rev: 5’-ccttccagccttatagggaac-3’	Eurofins	N/A

**Software and algorithms**		

R with R shiny package	r-project.org; shiny.rstudio.com	RRID:SCR_001905, RRID:SCR_001626
ImageJ	https://imagej.nih.gov/ij/	RRID: SCR_003070
MATLAB R2021b	The MathWorks	RRID: SCR_001622
OriginPro 2018G	OriginLab Corporation	RRID: SCR_014212
Clampfit 10.2	Molecular Devices	RRID: SCR_011323
Custom archieved	This manuscript	https://github.com/janadahlmanns/transporter_density/

### Resource availability

#### Lead contact

Further information and requests for resources and materials should be directed to and will be fulfilled by the Lead Contact, Dr. Christian Alzheimer (christian.alzheimer@fau.de).

#### Materials availability

This study did not generate any new materials or reagents.

### Experimental model and study participant details

#### Animals

Adult (2-5 months old) male wild type (wt) mice (with C57Bl/6J background) and transgenic mice expressing a dominant-negative mutant of activin receptor IB (dnActRIB) under the control of the CaMKII-α promoter^28^ were used for experiments. Animals were housed under standard conditions. All procedures were conducted according to the guidelines of Animal Protection Law of Germany and the European Communities Council Directive of November (1986/86/609/EEC), with approval of local government of Lower Franconia.

### Method details

#### Brain slice preparation

Horizontal hippocampal slices (350 μm thick) were prepared from adult mice using a vibratome (Leica VTS 1200 S, Leica Biosystems, Nußloch, Germany).^33^ Brain slices were cut in ice-cold sucrose-based artificial cerebrospinal fluid (aCSF, in mM: 75 sucrose, 3 KCl, 87 NaCl, 7 MgCl_2_, 0.5 CaCl_2_, 1.25 NaH_2_PO_4_, 25 NaHCO_3_, and 10 D-glucose). The first two slices per hemisphere, starting from the appearance of the complete hippocampal formation, were taken as dorsal hippocampus (DH), the last two slices per hemisphere still containing the hippocampal formation were taken as ventral hippocampus (VH). Slices were warmed to 35°C for 10 minutes in sucrose artificial cerebrospinal fluid (aCSF), and then stored at room temperature in low-Ca^2+^ and high-Mg^2+^ aCSF (in mM: 125 NaCl, 3 KCl, 1.25 NaH_2_PO_4_, 25 NaHCO_3_, 10 D-glucose, 1 CaCl_2_, 3 MgCl_2_, pH 7.4, gassed with 95% O_2_ and 5% CO_2_).

#### Electrophysiological recordings

After >1 h incubation at room temperature (RT), individual slices were transferred to a submerged recording chamber perfused with aCSF as above, except for physiological divalent cation concentrations (1.5 mM MgCl_2_ and 2.5 mM CaCl_2_) at 31 ± 1°C, gassed with 95% O_2_ - 5% CO_2_. The clear difference in hippocampal morphology between the dorsal and ventral pole precluded a ‘blinded’ approach regarding the location of the slice within the hippocampal formation. Recorded signals from visualized dorsal and ventral CA1 regions (upright microscope, Zeiss, Jena, Germany; equipped with Dodt gradient contrast) were low-pass filtered at 6 kHz (for whole-cell current-clamp recording) or 2 kHz (for field potential recording) and sampled at 20 kHz using a Multiclamp 700B amplifier in conjunction with Digidata 1440A interface and pClamp10 software (Molecular Devices, Sunnyvale, CA).

Whole-cell recordings of visually identified CA1 pyramidal cells were performed with glass pipets filled with (in mM) 135 K-gluconate, 5 HEPES, 3 MgCl_2_, 5 EGTA, 2 Na_2_ATP, 0.3 Na_3_GTP and 4 NaCl (pH 7.3). Electrode resistance with internal solution in bath medium was 3 to 5 MΩ before seal formation. After Gigaseal formation, whole-cell configuration was achieved by membrane rupture in voltage-clamp mode (V_h_ -70 mV). About 3 min later, membrane capacitance (Cm) and input resistance (R_N_) were measured using a ‘membrane test’ protocol delivering 10 mV biphasic pulses. Series resistance was between 5 to 20 MΩ. Voltage readings were corrected for liquid junction potential. R_N_ was re-examined after switching to current-clamp mode using hyperpolarizing pulses (5 mV) from membrane potential set to -70 mV by current injection. Depolarizing ramps (from 0 to 100 pA within 2 s) were used to elicit action potentials (APs) from resting membrane potential (RMP; Figure 1A) or -70 mV. Rheobase was determined as minimum current necessary to elicit AP during ramp depolarization. In the few cases where the standard depolarizing ramp failed to evoke APs from rest, we increased the rate of depolarizing current during the 2 s period until spiking appeared. Whereas threshold values from steeper ramps were included in the rheobase data set, quantification of number of APs per ramp was based solely on the discharge behavior during standard ramps. AP threshold was measured from first AP in ramps from rest, and computed from first time derivative of voltage as function of voltage. Spike threshold corresponded to maximum slope in this plot.^61^

Field postsynaptic potentials (fPSP) were recorded from CA1 stratum radiatum in brain slices from adult male mice with glass pipets filled with modified aCSF, in which NaHCO_3_ was replaced by HEPES (5 mM) to avoid pH change. A concentric platinum bipolar electrode was inserted into stratum radium to stimulate Schaffer collaterals (SC) using constant current pulses 0.1 ms wide. For long-term potentiation (LTP) and long-term depression (LTD) of the SC-CA1 synapse, stimuli were delivered at 0.1 Hz before and after tetanus, with stimulus strength adjusted individually to obtain 30% (LTP) or 50% (LTD) of the maximal fPSP amplitude/slope during stable baseline. LTP was induced by high frequency stimulation (HFS) at 100 Hz for 1 s, repeated once after 20 s. In some experiments, de-potentiation (DP) of HFS-LTP was examined by applying 5 Hz stimulation for 3 min, beginning 5 min after HFS.^38^ LTD was evoked by paired-pulse low-frequency stimulation (PP-LFS) at 1 Hz for 15 min, with paired stimuli 50 ms apart. Slopes were calculated from rising phase (20-80%) of fPSPs, normalized as percentage of baseline value, and pooled across experiments of the same group. Fiber volleys (FVs) were pharmacologically isolated by kynurenic acid (KA, 2 mM) and picrotoxin (100 μM) to block fast glutamatergic and GABAergic synaptic neurotransmission, respectively. Stimulating strength was chosen to obtain FV peak amplitudes of about 0.5 mV during 0.1 Hz baseline stimulation.

#### Enzyme-linked immunoabsorbent assay (ELISA)

Adult mice were sacrificed under isoflurane or sevoflurane anesthesia and brains were rapidly extracted and placed into ice-cold aCSF. Dorsal and ventral regions were cut from the insolated hippocampi, and put into lysis buffer (pH = 8.0, 0.32 M sucrose, 5 mM Tris-HCl, protease inhibitor cocktail from Sigma-Aldrich, Steinheim, Germany). The tissue was then mechanically broken down with a magnetic homogenizing ball for 20 s, centrifuged (13,000 x *g*, 4°C, 10 min, twice) and supernatant was collected. Activin A level was measured in the supernatant fraction according to manufacturer’s instructions with the Quantikine Activin A assay (Bio-techne GmbH, Wiesbaden-Nordenstadt, Germany).

#### RNA analysis

Brains from adult male dnActRIB mice were extracted as described for ELISA procedures in this manuscript. Dorsal and ventral hippocampi were prepared and separately stored at -80°C. RNA was then isolated according to manufacturer’s protocol (RNeasy Plus Universal Mini Kit; QIAGEN, Hilden, Germany), written into cDNA (High-Capacity cDNA Reverse Transcription Kit; Applied Biosystems, Germany), and then stored at -20°C. RNA levels were determined during quantitative real-time polymerase chain reactions (qPCR) according to manufacturer’s instructions (ABsolute QPCR Mix SYBR Green no ROX, Thermo Scientific, Germany) in realplex4 cycler (Eppendorf, Hamburg, Germany). Following primers were used (Eurofins Genomics, Ebersberg, Germany):

- dnActRIB (NM_007395.3, exon 6-7 and exon 7-8 deleted in the dnActRIB mutant,^28^ 130 bp); forward: 5’-tctaccataaccgccagagg-3’; reverse: 5’-aggtcctcctcggaaatcag-3’
- ActRIB (149 bp): forward: 5’-gtggggaccaaacgatacat-3’; reverse: 5’-tcatggactcctccagaattg-3’.
- TBP (146 bp); forward: 5’-gccaagagtgaagaacaatcc-3’, reverse: 5’-ccttccagccttatagggaac-3’.

Every biological sample was measured as a technical duplicate and then averaged. Then, ΔCq values were calculated by relating dnActRIB to TBP, and 2^-ΔΔCq^ values were calculated by normalizing values to the mRNA abundance of ActRIB in the DH of dnActRIB mice. Values were compared using an unpaired, two-tailed Student’s student’s t-test at α = 0.05.

#### Western blotting

Brains from adult male wt mice were extracted as described for ELISA experiments. Whole hippocampus was then prepared and homogenized by magnetically induced movement in lysis buffer (0.32 M sucrose, 5 mM Tris-HCl at pH 8.0, and protease inhibitor cocktail from Sigma Aldrich). Protein concentration was measured in a bicinchoninic acid (BCA) assay. After heating (95°C for 5 min), protein solution containing dithiothreitol (Sigma Aldrich) and sodium dodecyl sulfate running buffer was applied onto gels (10% TGX stain-free precast 298; Bio-Rad, Feldkirchen, Germany). Proteins were separated at 120 V for 1.5 h - 2 h and transferred onto polyvinylidenfluorid membranes (Bio-Rad). Prior to blotting (100 V, 30 min), membranes were activated in methanol (Carl Roth, Karlsruhe, Germany) for 30 s and then placed in transfer buffer (20% Methanol, 25 mM Tris-HCl, 192 mM glycine, pH 8.3). Blotting was performed at 100 V for 30 min. After washing in TBS-T (in mM: 10 Tris-HCl, 150 NaCl, 0.1 % Tween20), membranes were blocked with skim milk (5%, in TBS-T; Carl Roth, Germany). Membranes were incubated overnight at 4°C with primary antibody (recombinant anti-activin receptor IB/ALK4 antibody, ab109300, Abcam, rabbit, 1:1000 in TBS-T). After washing thrice with TBS-T, membranes were incubated for 1h at RT with secondary antibody (goat anti-rabbit IgG H&L coupled to horseradish peroxidase; ab6721, Abcam, 1:20.000 in TBS-T and 5 % milk). After washing thrice in TBS-T, protein content on membranes was visualized (ChemoStar Imager, Intas Science Imaging Instruments, Göttingen, Germany) using enhanced chemiluminescence (ECL) Western Blotting Substrates (Bio-Rad). Then, membranes were washed again thrice and incubated for 1.5 h at RT with monoclonal anti-β-actin-peroxidase antibody (A3854, Sigma Aldrich, 1:10.000 in TBS-T, 5 % silk milk) and imaging procedure was repeated as described above. Optical density of protein bands was corrected for background and related to β-actin. Measurements were performed with three technical replicates for each animal's DH and VH, which were then averaged. For statistical analysis, mean values of the biological replicates were averaged, and the resulting relative optical densities were compared between the DH and VH using a two-tailed, unpaired student’s t-tests at α = 0.05.

#### Hippocampus culture, immunocytochemistry and imaging

Preparation of hippocampal cultures was performed by the extraction of hippocampus tissue from wt mice (P2-P5). Tissue was then trypsin-digested, mechanically triturated, centrifugated, and cells were plated onto cover slips for further use.^33^ In treatment groups at DIV 20-25, activin A was added (25 - 50 ng/ml, cat. no. 338-AC; Bio-Techne, Wiesbaden, Germany) for incubation (24 h) before the staining procedure. After washing briefly with PBS, cells were fixated in paraformaldehyde (4 %) for 20 min and washed again with PBS (thrice, 10 min). After incubation with blocking buffer (containing 5 % normal donkey serum, 1 % BSA, PBS, and 0.5 % Triton-X-100) cells were incubated with primary antibodies against VGLUT1 (rabbit monoclonal recombinant IgG, cat. no. 135 308, use 1:1000; Synaptic Systems, Göttingen, Germany) and MAP2 (guinea pig polyclonal antiserum, cat. no. 188 004, 1:1000, Synaptic Systems) for 2 h at RT. After washing with PBS (thrice, 10 min), cells were incubated with secondary antibodies (in blocking solution: donkey-anti-guinea pig Cy3, use 1:1000, A21206, Thermo Fisher Scientific; donkey-anti-rabbit Alexa488, use 1:1000, A-21206, Thermo Fisher Scientific) for 1 h in the dark at RT. After washing with PBS (thrice, 10 min), coverslips were mounted with Roti®Mount Fluor Care DAPI (Carl Roth, Karlsruhe, Germany). Imaging was then performed using a confocal LSM780 (Zeiss, Jena, Germany) and a Plan-Apochromat 63x/1.40 Oil DIC objective. Images were converted to the file format .tif using Zen Blue (Zeiss) for further analysis.

The resulting images were analyzed with a custom R shiny (RRID:SCR_001626) script. A user, blinded to the experimental condition, chose fluorescence intensity thresholds for the red (MAP2) and green (VGLUT1) channel. Only above-threshold pixels were analyzed to exclude faint background fluorescence, stemming from general imaging noise and emission outside the imaging plane. We calculated the total fluorescence intensity for the VGLUT1 channel. For normalization of fluorescence area, the resulting values were normalized to MAP2 fluorescence area in each image to account for varying amounts of dendrites captured by the fields of view. Experimental groups were analyzed using a one-way ANOVA followed by Tukey’s post-hoc test (alpha = 0.05) and shown as mean ± SEM.

### Quantification and statistical analysis

Electrophysiological data analysis was realized using Clampfit 10.2 (Molecular Device) and MATLAB R2021b (Natick, MA, USA). Data are presented as mean ± SEM (standard error of the mean). OriginPro 2018G (OriginLab Corporation, MA, USA) was used for statistics and figures. Imaging data was analyzed using the programming language R (RRID: SCR_001905). Statistical comparisons of data were performed using one-way ANOVA or Student’s student's t test, as appropriate and stated in the respective method part. Significance was assumed for p < 0.05. Inside the figures, statistical significance was shown with asterisks (p < 0.05 ∗, p < 0.01 ∗∗, p < 0.001 ∗∗∗).

## Data Availability

• Data reported in this paper will be shared by the lead contact upon request. • Custom generated R code for the immunocytochemical data analysis is stored under a publicly available GitHub repository (https://github.com/janadahlmanns/transporter_density/). • Any additional information required to reanalyze the data reported in this publication are available from the lead contact upon request.
